# Supplementary material for: Content-rich biological network constructed by mining PubMed abstracts
Source: BMC Bioinformatics. 2004 Oct 8;5:147. doi: 10.1186/1471-2105-5-147 (PMC528731; doi:10.1186/1471-2105-5-147)
Supplement: Additional File 2 — The original results of the above study (non-essential files are deleted to keep the file size under the limit set by BMC bioinformatics). [file 1471-2105-5-147-S2.bz2 › chilibotAdditionalFile2/dip10/140ID7739537E499/html/E2F2_RB1.html]

 


 **E2F2** and **RB1** 
  
Found 31 abstracts in PubMed, retrieved 10.  
 

 What does Google say? 
 PDF only 
| .edu only 

---

- Mol Cell Biol, 2003   **Constitutive E2F1 Overexpression Delays Endochondral Bone Formation by Inhibiting Chondrocyte Differentiation.** .
  Longitudinal bone growth results from endochondral ossification, a process that requires proliferation and differentiation of chondrocytes.
  It has been shown that proper endochondral bone formation is critically dependent on the retinoblastoma **[RB1]** family members p107 and p130.
  However, the precise functional roles played by individual E2F proteins remain poorly understood.
  Using both constitutive and conditional E2F1 transgenic mice, we show that ubiquitous transgene driven expression of E2F1 during embryonic development results in a dwarf phenotype and significantly reduced postnatal viability.
  Overexpression of E2F1 disturbs chondrocyte maturation, resulting in delayed endochondral ossification, which is characterized by reduced hypertrophic zones and disorganized growth plates.
  Employing the chondrogenic cell line ATDC5, we investigated the effects of enforced E2F expression on the different phases of chondrocyte maturation that are normally required for endochondral ossification.
  Ectopic E2F1 expression strongly inhibits early and late phase differentiation of ATDC5 cells, accompanied by diminished cartilage nodule formation as well as decreased type II collagen, type X collagen, and aggrecan gene expression.
  In contrast, overexpression of **E2F2** or E2F3a results in only a marginal delay of chondrocyte maturation, and increased E2F4 levels have no effect.
  These data are consistent with the notion that E2F1 is a regulator of chondrocyte differentiation.

  - Genes Dev, 2002   **Structural basis for the recognition of the E2F transactivation domain by the retinoblastoma **[RB1]** tumor suppressor.** .
    Repression of E2F transcription activity by the retinoblastoma **[RB1]** Rb tumor suppressor through its interaction with the transactivation domain of the E2F transcription factor is one of the central features of G1 S arrest in the mammalian cell cycle.
    Deregulation of the Rb E2F interaction results in hyperproliferation, lack of differentiation, and apoptosis, and can lead to cancer.
    The 2.2 A crystal structure of the Rb pocket complexed with an 18 residue transactivation domain peptide of E2F 2 **[E2F2]** reveals that the boomerang shaped peptide binds to the highly conserved interface between the A box and the B box of the Rb pocket in a bipartite manner.
    The N terminal segment of the E2F 2 **[E2F2]** peptide in an extended beta strand like structure interacts with helices from the conserved groove at the A B interface.
    . . . the C terminal segment, which contains one 3 10 helix, binds to a groove mainly formed by A box helices.
    The flexibility in the middle of the E2F 2 **[E2F2]** peptide is essential for the tight association of E2F to the Rb pocket.
    The binding of Rb to the E2F 2 **[E2F2]** peptide conceals several conserved residues that are crucial for transcription activation of E2F.
    We provide the structural basis for the Rb mediated repression of E2F transcription activity without the requirement of histone modifying enzymes.

    - Mol Cancer Ther, 2002   Several studies have identified silibinin as an anticarcinogenic agent.
      Recently, we showed that silibinin inhibits cell growth via G1 arrest, leading to differentiation of androgen dependent human prostate carcinoma LNCaP cells .
      Here, we extend this study to assess the effect of silibinin on total retinoblastoma **[RB1]** protein levels and its phosphorylation status, levels of E2F family members, and Rb E2F binding in LNCaP cells.
      Compared with controls, silibinin resulted in an increase in total Rb levels that was largely attributable to an increase in unphosphorylated Rb .
      This effect of silibinin was mainly attributable to a large decrease in the amount of Rb phosphorylated at specific serine sites.
      In other studies, silibinin showed a moderate effect on E2F1 but up to 98 and 90% decreases in **E2F2** and E2F3 protein levels, respectively.
      Silibinin treatments also resulted in an increase in the amount of Rb binding to E2F1 , **E2F2** , and E2F3 .
      Cyclin dependent kinases , together with their catalytic subunit cyclins, phosphorylate Rb, which makes transcription factor E2Fs free from Rb E2F complexes, resulting in cell growth and proliferation.
      Conversely, CDK inhibitors inhibit this phosphorylation, maintaining E2Fs bound to Rb, which causes growth inhibition.
      On the basis of our data showing that silibinin induces both unphosphorylated Rb levels and Rb E2F binding, we also assessed its effect on upstream cell cycle regulators.
      Silibinin treated cells showed up to 2.4 and 3.6 fold increases in Cip1 p21 and Kip1 p27 levels, respectively, and a decrease in CDK2 , CDK4 , and cyclin D1 .
      Consistent with these results, silibinin showed both G1 arrest and growth inhibition.
      Together, these findings identify modulation of Rb levels and its phosphorylation status as a molecular mechanism of silibinin induced neuroendocrine differentiation of human prostate carcinoma LNCaP cells and suggest that this could be a novel approach for prostate cancer prevention by silibinin.

      - Cancer Res, 2002   **Unbiased analysis of RB mediated transcriptional repression identifies novel targets and distinctions from E2F action.** .
        The retinoblastoma **[RB1]** tumor suppressor, RB, is thought to inhibit cell cycle progression through transcriptional repression.
        E2F regulated genes have been viewed as presumptive targets of RB mediated repression.
        However, we found that specific E2F targets were not regulated in a consistent manner by the action of a RB allele that is refractory to cyclin dependent kinase cyclin mediated phosphorylation PSM RB when compared with **E2F2** overproduction.
        Therefore, we used Affymetrix GeneChips as an unbiased approach to identify RB targets.
        We found that expression of PSM RB significantly attenuates >200 targets, the majority of which are involved in cell cycle control DNA replication or G2 M , DNA repair, or transcription chromatin structure.
        The observed repression was due to the action of RB and not merely a manifestation of altered cell cycle distribution.
        Additionally, the majority of RB repression targets were confirmed through the blockade of endogenous RB phosphorylation via p16ink4a overexpression.
        Thus, these results have utility in assigning RB pathway activation in more complex systems of cell cycle inhibition e.g., mitogen withdrawal, senescence, or DNA damage checkpoint .
        As expected, a significant fraction of RB repressed genes have promoters that are bound regulated by E2F family members.
        However, targets were identified that are distinct from genes known to be stimulated by overexpression of specific E2F proteins.
        Moreover, the relative action of RB versus **E2F2** overexpression on specific genes demonstrates that a simple opposition model does not explain the relative contribution of RB to gene regulation.
        Thus, this study provides the first unbiased description of RB repressed genes, thereby delineating new aspects of RB mediated transcriptional control and novel targets involved in diverse cellular processes.

        - Exp Cell Res, 2002   **Active RB elicits late G1 S inhibition.** .
          The retinoblastoma **[RB1]** tumor suppressor protein RB is activated dephosphorylated to mediate cell cycle inhibition in response to antimitogenic signals.
          To elucidate the mode of RB action at this critical transition, we utilized cell lines that can be induced to express a constitutively active allele of RB PSM RB .
          As expected, induction of PSM RB, but not wild type protein WT , inhibited progression into S phase.
          It has been well documented that active RB inhibits E2F reporter activity, and this observation was confirmed upon induction of PSM RB.
          Additionally, active RB inhibited E2F 2 **[E2F2]** mediated stimulation of cyclin E.
          By contrast, PSM RB did not affect the mRNA or protein levels of endogenous cyclin E when mediating cell cycle inhibition.
          Similarly, there was no observable effect on cyclin E protein levels when p16ink4a was utilized to activate endogenous RB.
          CDK2 cyclin E complex formation was not disrupted and cyclin E associated kinase activity was retained in the presence of PSM RB.
          Additionally, centrosome duplication, a CDK2 cyclin E dependent event, was not altered in the presence of active RB.
          Together, these data indicate that active RB does not block the G1 S transition through inhibition of cyclin E expression or activity.
          In contrast, PSM RB leads to a dramatic reduction in cyclin A protein levels by coordinate transcriptional repression and degradation.
          This attenuation of cyclin A protein correlates with cell cycle inhibition.
          These studies indicate that RB inhibits cell cycle progression by targeting CDK2 cyclin A dependent events at the G1 S transition to inhibit cell cycle progression.

          - Invest Ophthalmol Vis Sci, 2002   **Unique roles for E2F1 in the mouse lens in the absence of functional pRB proteins.** .
            PURPOSE Normal lens fiber cell differentiation requires functional retinoblastoma **[RB1]** protein pRB , because inactivation of this protein results in proliferation and apoptosis in normally postmitotic, differentiating fiber cells.
            Loss of either E2F1 or 3 can partially rescue the lens phenotype in Rb deficient mice, implying that these E2Fs may have specific targets in this system.
            The purpose of this study was to determine what unique role E2F1 may play.
            METHODS Expression of E2F family members and target genes was analyzed in the lenses of nontransgenic, E2F1 null, alphaAE7;E2F1 sufficient.
            and alphaAE7;E2F1 null mice by in situ hybridization, Northern blot analysis, and RT PCR.
            RESULTS In lenses of E2F1 null mice, there was no change in the expression of E2F 2 **[E2F2]** to 5 or their target genes, compared with E2F1 sufficient mice.
            However, in the lens of alphaAE7 mice where pRB proteins are inactivated, expression of **E2F2** and 3a was increased.
            The E2F3a increase, but not that of **E2F2**, was dependent on E2F1.
            Expression of E2F target genes was increased with expression of E7 and expression of one of these, p19ARF, was E2F1 dependent.
            CONCLUSIONS Although in the normal lens there do not appear to be unique roles for E2F1 that cannot be fulfilled by other E2F family members, in the absence of functional pRB proteins, E2F1 is specifically responsible for the increased expression of E2F3a and p19ARF.
            These findings suggest that E2F1 may be the preferred E2F regulating these target genes in the normal lens.

            - Nature, 2001   **The E2F1 3 transcription factors are essential for cellular proliferation.** .
              The retinoblastoma **[RB1]** tumour suppressor Rb pathway is believed to have a critical role in the control of cellular proliferation by regulating E2F activities.
              E2F1, **E2F2** and E2F3 belong to a subclass of E2F factors thought to act as transcriptional activators important for progression through the G1 S transition.
              Here we show, by taking a conditional gene targeting approach, that the combined loss of these three E2F factors severely affects E2F target expression and completely abolishes the ability of mouse embryonic fibroblasts to enter S phase, progress through mitosis and proliferate.
              Loss of E2F function results in an elevation of p21Cip1 protein, leading to a decrease in cyclin dependent kinase activity and Rb phosphorylation.
              These findings suggest a function for this subclass of E2F transcriptional activators in a positive feedback loop, through down modulation of p21Cip1, that leads to the inactivation of Rb dependent repression and S phase entry.
              By targeting the entire subclass of E2F transcriptional activators we provide direct genetic evidence for their essential role in cell cycle progression, proliferation and development.

              - Genes Dev, 2001   **E2F3 contributes both to the inappropriate proliferation and to the apoptosis arising in Rb mutant embryos.** .
                The E2F transcription factors are thought to be key downstream targets of the retinoblastoma **[RB1]** protein pRB tumor suppressor.
                It is widely believed that E2F1, **E2F2**, and E2F3 can all activate cellular proliferation but that E2F1 is the specific inducer of apoptosis.
                Here we show that the E2f3 mutation completely suppresses both the inappropriate proliferation and the p53 dependent apoptosis arising in the Rb mutant embryos.
                Through the analysis of Rb ;E2f3 embryos, we have been able to separate E2F3 s role in the induction of apoptosis from its ability to induce proliferation.
                Thus, contrary to the prevailing view of E2F action, E2F3 makes a major contribution to the apoptosis resulting from pRB loss.

                - Genes Dev, 2001   **E2Fs regulate the expression of genes involved in differentiation, development, proliferation, and apoptosis.** .
                  The retinoblastoma **[RB1]** protein pRB and its two relatives, p107 and p130, regulate development and cell proliferation in part by inhibiting the activity of E2F regulated promoters.
                  We have used high density oligonucleotide arrays to identify genes in which expression changed in response to activation of E2F1, **E2F2**, and E2F3.
                  We show that the E2Fs control the expression of several genes that are involved in cell proliferation.
                  We also show that the E2Fs regulate a number of genes involved in apoptosis, differentiation, and development.
                  These results provide possible genetic explanations to the variety of phenotypes observed as a consequence of a deregulated pRB E2F pathway.

                  - Invest Ophthalmol Vis Sci, 2000   **Expression patterns of retinoblastoma **[RB1]** and E2F family proteins during corneal development.** .
                    PURPOSE To determine the expression patterns of the retinoblastoma **[RB1]** protein and the E2F transcription factor families in limbal and corneal epithelia and in corneal keratocytes in situ during corneal development and differentiation.
                    METHODS Retinoblastoma **[RB1]** protein pRb and its family members p107 and p130.
                    E2F , and 4, members of the E2F family of transcription factors.
                    and Ki67, a marker of actively cycling cells, were localized by indirect immunofluorescence microscopy, in corneas of neonatal, juvenile, and adult rats.
                    Presence of mRNA for pRb, p107, p130, and E2F types 1 to 5 in adult corneal epithelium was determined by reverse transcription polymerase chain reaction.
                    RESULTS mRNA for all members of pRb and E2F families was present in adult corneal epithelium.
                    The greatest number of Ki67 positive corneal and limbal epithelial cells were present at days 13 to 19, and Ki67 positive stromal keratocytes at day 2.
                    pRb and E2F 2 **[E2F2]** were localized to all cells in neonatal, juvenile, and adult corneas.
                    With age, p130 localization became more intense and nuclear in stromal keratocytes and suprabasal cells of corneal and limbal epithelia.
                    p107, initially nuclear in limbal and corneal epithelia, became increasingly cytoplasmic in corneal epithelium.
                    E2F 1 was initially nuclear in keratocytes and diminished after day 10.
                    E2F 1 was localized in the basal cell layer of limbal and corneal epithelia after day 10.
                    E2F4 was always nuclear in limbal epithelium and cytoplasmic in corneal epithelium.
                    CONCLUSIONS Expression patterns of pRb and E2F family proteins vary with corneal cell differentiation, but are most apparent with p130 and p107.
                    Nuclear localization of p130 appears to correlate with terminal differentiation in epithelium and entrance into a quiescent state by keratocytes.
                    In contrast, p107 is nuclear in the undifferentiated limbal basal cells and is cytoplasmic in the remainder of the corneal epithelial cells.
